# 

**Published:** 1881-02-20

**Authors:** 


### EDITORIALS AND MISCELLANEOUS.

### *EDITORIAL NOTICES.*

*Maltine in Phthisis.*—Examine carefully Reed & Carnrick's preparations on inside back cover page.

*Trommer's Extract of Malt.*—The advertisement of this excellent preparation should be examined—on first inside cover page—especially useful in certain forms of dyspepsia.

*Johnston's Fluid Beef*—Don't fail to examine the advertisement of the above article. We have used it, and can recommend it as a superior preparation.

*Powell's Manufacturing Co., Baltimore, Md.*—See the insert of the above Co., in this Journal. We have reason to believe that their prepations are excellent. We are particularly pleased with their Beef, Cod Liver Oil and Pepsin.

*The Popular Science Monthly for January*, is a number of great interest, sustaining its high character as a popular Scientific Journal. It is published by D. Appleton & Co., 1, 2 and 3 Broad St., New York. Subscription $5 per annum.

*Sunday Gazette.*—This is a large weekly newspaper, published in Atlanta, and very popular with the citizens of the city and the reading public generally. We can recommend it as an excellent Political and Miscellaneous Family paper. See the advertisement in this journal.

*The Southern Medical College—Atlanta.*—The Commencement exercises of the above School will take place on the evening of the 3d of March. The occasion will doubtless be an interesting one. The Commencement Address will be delivered by Rev. Mr. Evans, of Atlanta, at DeGive's Opera House, and the Diplomas will be presented by Prof. Powell, the President of the Board and Faculty. A valedictory will be delivered by a member of the class, and other exercises of an attractive character will take place. In our next issue we will give a more particular account, embracing a list of the graduates and the entire programme. Resident Physicians and Medical men visiting the city, and all parties interested, are invited to be present. W.

### *RECEIPTED.*

[Receipts not acknowledged privately are entered here.]

1880—R Inge, J A Ardry, J L Dement, R J McMullen, G B Battle, Wm Sellers, J B Murfree, J S Miller, J G McCrary, R A Sills, H H Bate.

1881—E A Cox, R L Hinton, P Taylor, Thos A Boggan, Robert James, J W Patton, R J Walker, J N Wardworth, J G Montgomery, J H Wyly, F Courtney, C B Sewell, J O Sanders, J H Nance, A C Simonton, B F Duke, J A Williams, D B Searcy, Lockwood Allison, R F Mathews, J N West, R B Shelby, A B McWhorter, M F Alford, W F Funderburg, J B Bailey, M B Pollard, E B Young, A G Smythe, S W Eaton, J R Green, A Atkinson, C M Church.

